# Formulation of Epoxy Prepregs, Synthesization Parameters, and Resin Impregnation Approaches—A Comprehensive Review

**DOI:** 10.3390/polym16233326

**Published:** 2024-11-27

**Authors:** Yashoda Somarathna, Madhubhashitha Herath, Jayantha Epaarachchi, Md Mainul Islam

**Affiliations:** 1 Centre for Future Materials, University of Southern Queensland, Toowoomba 4350, Australia; yashoda.somarathna@unisq.edu.au (Y.S.); jayantha.epaarachchi@unisq.edu.au (J.E.); 2 School of Mechanical and Electrical Engineering, Faculty of Health Engineering and Sciences, University of Southern Queensland, Toowoomba 4350, Australia; 3 School of Engineering, University of Hull, Hull HU6 7RX, UK; madhubhashitha@uwu.ac.lk

**Keywords:** epoxy prepregs, resin formulation, viscosity, tack, B-stage

## Abstract

Prepregs are resin-impregnated, expensive composites mainly limited to high-end applications within the aeronautical, defense, automotive, and energy sectors. Prepreg technology is mainly protected by trade secrets, resulting in limited studies on prepreg resin matrix development and recent advancements. Three key parameters for epoxy resin matrix development including B-staging, viscosity, and tackiness, and their control strategies are discussed in detail. The B-stage is defined as the partially cured stage of epoxy prepregs and is extremely important for prepreg layup, pot life, and final performances. The three key parameters are interrelated and accurately controlled, and, hence, resin development plays a huge role in the prepreg development process. This review also discusses the measuring techniques of the parameters in detail. Based on the resin impregnation techniques and B-stage control, two approaches are proposed to develop the prepreg resin formulations: conventional resin impregnation and viscosity-controlled resin impregnation. The approaches would be extremely useful, especially for advancing beyond the existing prepreg applications and developing smart materials and functional composites through advanced resin modification strategies.

## 1. Introduction

Nowadays, fiber-reinforced polymer composites are widely used in manufacturing industries, and, as a result, the demand for traditional bulky metallic materials such as steel and metal alloys has gradually decreased [1,2]. Due to this reason, material scientists have developed a novel composite material by pre-impregnating resin in a fiber fabric which is commonly known as a prepreg and has been branded as a viable raw material for many advanced applications [1,3,4,5]. Commercial prepregs are available in rolls and can be utilized for the direct layups of complex molds without handling any liquid resins [6,7,8]. Most importantly, prepregs reduce the chance of having a poor resin distribution and, ultimately, enhance the quality and consistency of the components [7]. The resin used in prepregs is either pre-catalyzed or B-staged in order to reduce the cure time when it is molded during the layup [9,10,11]. Most importantly, prepregs can be used in applications where excellent performance, quality, and consistency are required in the final product, such as aerospace, defense, and automotive components [12,13,14].

According to Duhovic et al., the first prepregs were developed in the late 1980s by impregnating thermoplastic matrices into fibers [15]. However, in a recent review conducted by Lukaszewicz et al., on automated prepreg layup technology, the inception of the prepreg history goes back to as early as 1970s, during which commercial carbon fibers became available [16]. Further improvements in the automated tape layup (ATL) and manual fabrication of prepregs took place during the 1980s [16,17,18,19]. An insight into historical prepreg developments which are found in the literature is highlighted in Table 1.

Most of the reviews published on prepreg development during the last decade and their main objectives are summarized in Table 2. Although there are numerous reviews conducted on prepregs, most of the reviews are mainly focused on post-curing, prepreg layup and property improvements, prepreg defects, and prepreg testing.

According to Table 2, three reviews are focused on the common manufacturing techniques and post-curing methods of prepregs including vacuum-bag-only (VBO) and out-of-autoclave (OOA) techniques [13,14,24]. Centea et al. have conducted a recent review on the material properties, process phenomena, and manufacturing considerations of OOA prepregs, especially for the aerospace industry [13]. The out-of-autoclave technique has gained great attention during the last decade as it brings autoclave quality parts but with a reduced cost and environmental impact, and also enables using diversified equipment including conventional ovens, heating blankets, etc. Further, the OOA technique can be used with less expensive and lightweight cores, and, as a result, it can eliminate some of the major autoclave-induced defects such as honeycomb core crush. When highlighting the resin properties, the authors have mentioned that most of the published work on VBO prepregs employed commercially available prepregs [13]. A similar review has been conducted by Shaik et al., focusing on the OOA prepregs for aerospace component manufacturing and highlighting the cost optimization and improvement of cycle time [14]. Ekuase et al. have conducted a review focusing more on OOA processing techniques for a wide range of applications [24]. The review summarizes some of the common OOA processing techniques including vacuum-bag-only, resin transfer molding, vacuum-assisted resin transfer molding, quickstep curing, Seeman composite resin infusion molding process, resin film infusion, and resin infusion under double flexible tooling.

Several recent reviews have been published highlighting the prepreg defects, the importance of quality measurements, and the involvement of nanotechnology to overcome the delamination of prepregs [25,27,28]. A critical review conducted by Hassan et al. on manufacturing defects highlighted the strong links between the manufacturing defects and sub-processes including the laying up, bagging, and curing of complex-shaped laminates for aircraft structures [25]. Another review conducted by the same group identified the possible defect-related factors during the process starting from the layup to curing process [28]. In the same review, the author concluded that the vacuum-bagging process is the most critical process that hugely contributes towards the void content and resin accumulation. However, there is no indication of the effect of the resin composition of prepregs on the discussed defects. The involvement of nanomaterials as a possible solution for the delamination effect and the common challenges for manufacturing nanoengineered prepregs are briefly discussed in a recent review conducted by Islam et al. [27]. As claimed by the authors, mixing nanomaterials with resin has been a common practice and the main challenges include maintaining resin viscosity during impregnation and avoiding particle agglomeration. However, information on the use of nanomaterials for resin matrix development in prepregs and their effect on resin viscosity is unclear. Finally, the authors have concluded that the addition of a nanolayer between the prepreg layers may have a significant effect in lowering the delamination effect. Björnsson et al. have emphasized the challenges of the automated layup process of prepregs in their review and highlighted that providing comprehensive solutions for material handling could be hard due to different material properties [26].

A very informative review of the prepreg tack and its underlying mechanisms and tack-measuring techniques has been conducted by Budelmann et al. [29]. The authors reported that the time–temperature superposition principle can be used to describe the relationship between the viscosity and tack of the resin matrix in prepregs. This is extremely useful for the development of the resin matrix for prepregs. Another review conducted by Jiang et al. have highlighted quality control strategies available for epoxy-based prepregs [30]. In this review, the use of near-infrared (NIR) spectroscopy and micro-CT (computed tomography) as quality control analysis techniques of prepregs have been highlighted.

Based on the recent reviews published on prepregs, it is important to notice that none of these studies are focused on resin matrix properties and development for prepregs and overlook prepreg development steps, which would be useful when developing new prepregs with improved properties. Moreover, the disclosure of the chemical composition has been restricted by most of the prepreg manufacturers; therefore, the available literature on prepreg components and chemical formulation is very scarce. The available literature on prepreg resin formulation development and their curing conditions is reviewed under Section 3.

**Figure 1:**
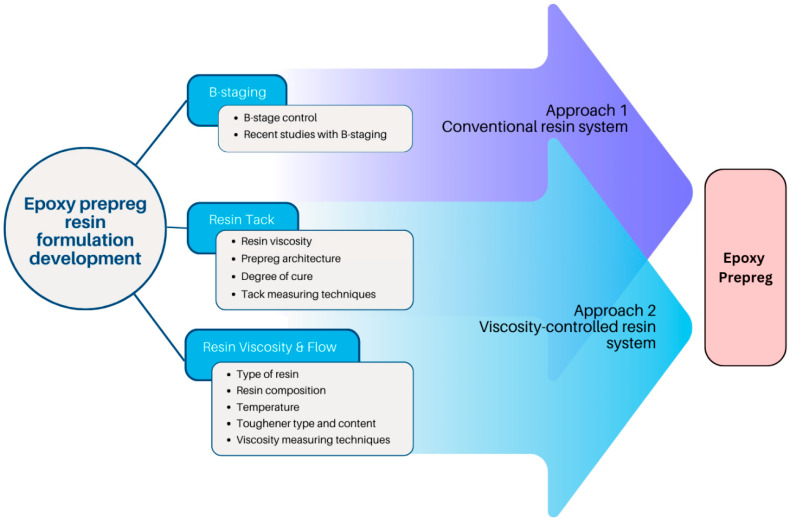
Overview of the review.

As described in the overview shown in Figure 1, this review mainly focuses on the epoxy resin matrix development parameters and their effects on the prepreg development process. The B-staging, resin tack, and resin viscosity are considered the most important epoxy resin parameters, and the control of the same is discussed in detail in Section 3. After reviewing the limited number of studies on resin matrix development, two approaches have been identified for prepreg resin impregnation, namely, the conventional resin impregnation and the viscosity-controlled resin impregnation. The differences between these two impregnation approaches along with the current challenges in epoxy prepreg development are discussed in Section 4. Moreover, the prepreg history, applications, and current prepreg market highlights are also discussed briefly in Section 2. As this study compiles recently published information mainly on epoxy resins for prepregs, this review would be extremely useful for the further modification of the prepreg resin and the use of the modified resin for smart material development.

## 2. An Insight into Current Prepreg Applications and Commercial Prepreg Manufacturing Market

### 2.1. Current Prepreg Applications

**Figure 2:**
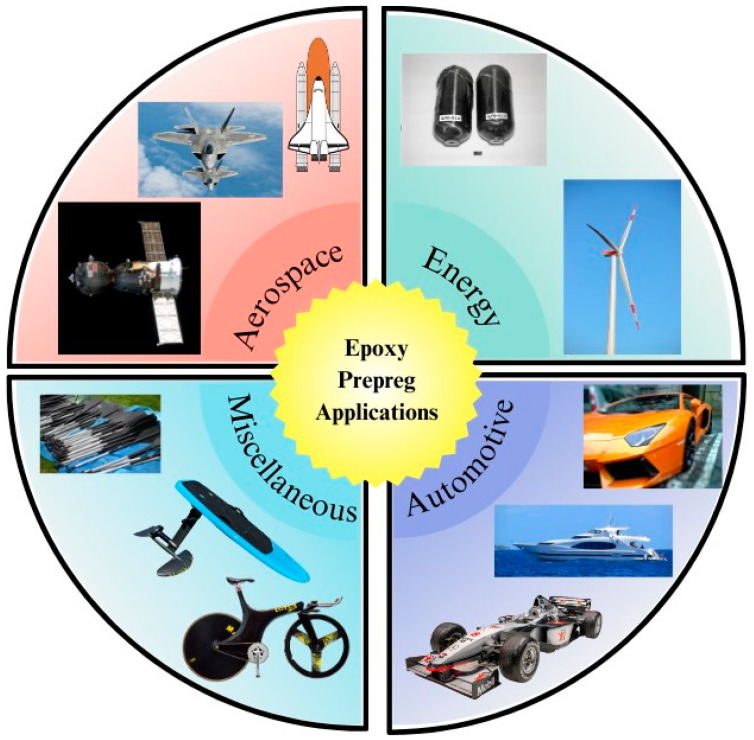
Overview of prepreg applications.

Owing to the excellent mechanical properties and light weight of prepregs, commercial prepreg manufacturing has increased rapidly during the last few decades. Compared to many other fiber-reinforced composite manufacturing processes, the use of prepregs in manufacturing is confined to high-performance components in aerospace, defense, luxury automotive, sporting equipment, and wind turbine manufacturing [3,31,32,33,34,35,36,37]. An overview of the main industrial sectors that use prepregs is shown in Figure 2.

It is important to highlight the significant increase in composite usage in the aerospace industry during the past few decades [3,31,36]. It is reported that the usage of composite materials especially in combat aircrafts havesignificantly increased from about 2 percent (by structural weight) to 25percent [36]. The article published by Setlak et al. highlighted the significant increase in the utilization of composite materials for the manufacturing of modern aircraft such as Airbus A-380, Boeing B-787, and Lockheed Martin F-35 from 2008 to 2019 [36,38]. It is further found that more than 50% of the total materials used for manufacturing modern Airbus and Boeing aircraft originated from prepregs [39,40]. Aircraft manufacturers often use automated tape layup (ATL) tools to produce large aircraft parts using epoxy prepregs. The tool head is multifunctional, thus enabling it to remove the backing paper from the prepreg tape, and the layup, cut the material from a precise location, and begin the same process from the next position [40].

Owing to the complex structure of wind turbines, prepreg technology has been used to produce various sizes of wind turbine blades by several major wind turbine producers in the world [41,42,43]. In early days, wind turbine blades were mainly produced through wet layup and wet winding methods [42]. However, the current trend is mostly towards hand layup prepregs and automated technologies such as automated tape layup (ATL) or automated fiber placement (AFP), resulting in very high-quality wind turbine blades [42]. Autoclave technology is often proven to be a better method to produce wind turbine blades with excellent structural properties [33].

One of the biggest restraints of using CFRP materials with thermoset resins in the automobile manufacturing process is the longer curing time, which ultimately limits the faster production process. However, this can be avoided by using prepregs along with an out-of-autoclave (OOA) curing process to generate faster and quality production processes. For the first time in 2014, the Mitsubishi Rayon Corporation in Japan produced decklid inner and outer panels for their supercar, Nissan GT-R, using prepregs and found out that the car’s trunk aesthetic has been increased, and, most importantly, with a 40% reduction of mass [44]. Lee et al. examined the feasibility of the vacuum-assisted prepreg compression molding (VA-PCM) technique to produce CFRP roof plates for automobiles [45]. Although VA-PCM involves a higher material cost when compared to conventional PCM, the panels produced by VA-PCM have few defects. The well-known Formula 1 car manufacturers have used prepreg technology to produce major body parts, which could be about 80% of the total volume of a car [35]. The replacement of the hood and roof parts for the sports car Corvette Stingray from epoxy-based carbon fiber prepregs has gained several advantages including a reduction in mass (about 50%), cycle time (66%), direct part cost (30%), and consumable cost (75%) without losing mechanical properties [46].

**Figure 3:**
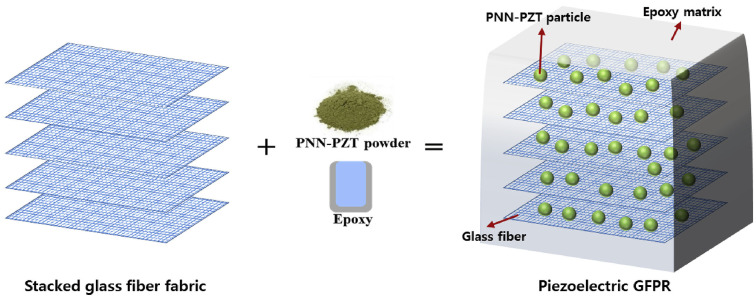
Schematic illustration of the preparation of piezoelectric GFRP for smart sensor applications. Reproduced with permission [47]. Copyright © 2018 Elsevier.

With the recent advances in smart materials, researchers have tried to integrate prepregs with some of the smart applications. Although most of these applications are still at the research level, it is worth highlighting some of them. Hwang et al. have developed piezoelectric GFRP (glass-fiber-reinforced polymer) prepregs by simply mixing piezoelectric powder (Pb(Ni_1/3_Nb_2/3_)O_3_-Pb(Zr, Ti)O_3_) (PNN-PZT) with epoxy resin, impregnated into glass fibers [47]. These composites can function as impact sensors and a summary of their process is shown in Figure 3. Although they have used an epoxy resin that requires a two-stage curing cycle and have fabricated the composites using the autoclave process, the name and grade of the epoxy or curing agent were not mentioned.

**Figure 4:**
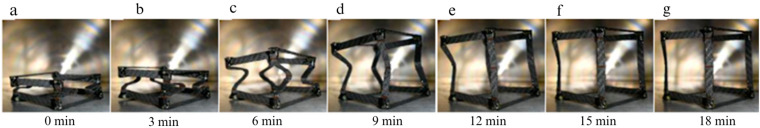
Shape recovery steps of cubic-shaped deployable structures made from shape memory polymer prepregs (from **a**–**g**). Reproduced with permission [52]. Copyright © 2019 The Institution of Engineers, Sri Lanka.

A group of researchers has developed shape-memory polymer prepregs by simply integrating shape-memory polymer powders between commercially available prepregs mainly for aeronautical structures [48,49,50,51]. A study conducted by Herath et al. on shape-memory polymer composites highlighted the use of shape-memory polymer prepregs for deployable space habitats as depicted in Figure 4 [51,52].

Nanoparticles such as graphene nanoplates, graphene oxides, and carbon nanotubes are used with prepregs to produce smart fiber-reinforced composites for various applications including wearable devices, machine tools, aircraft parts, sensors, etc. [53,54,55,56,57]. However, the availability of information on nanoengineered prepregs and smart prepregs is extremely limited and the practical use is still unclear.

### 2.2. Prepreg Market Highlight

**Figure 5:**
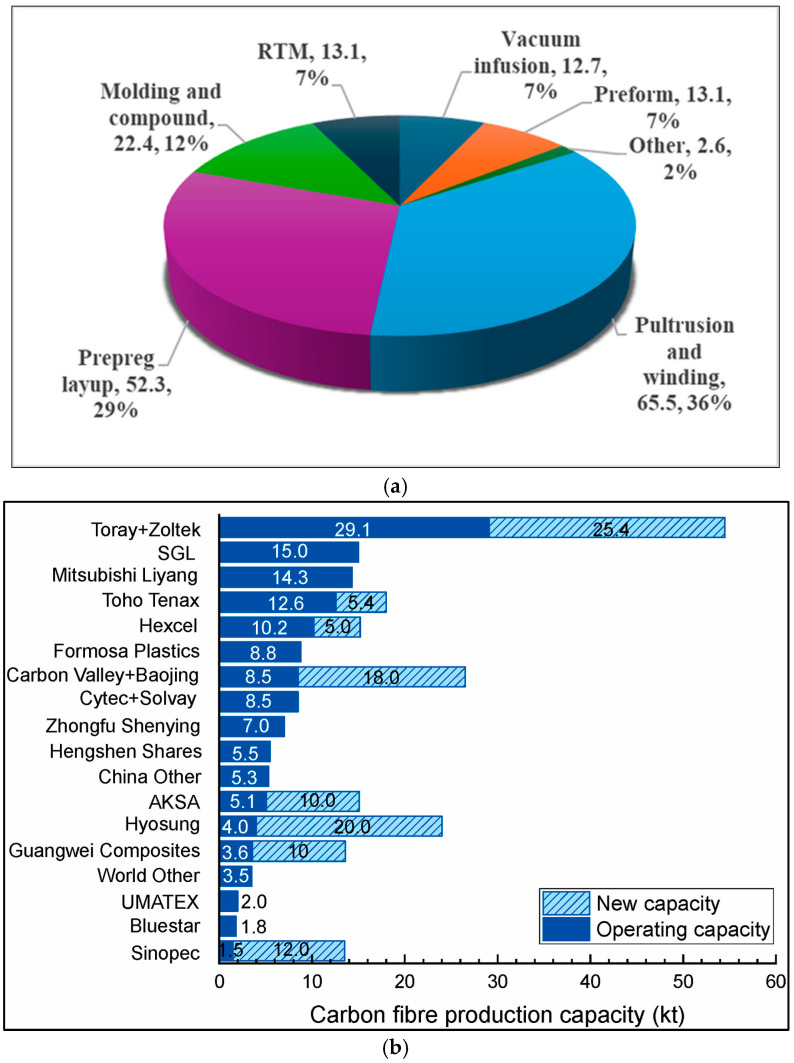
Market overview showing (**a**) CFRP production by process (in kilo-tons) and (**b**) main prepreg manufacturers in the world. Reproduced with permission [1]. Copyright © 2023 Elsevier.

The recent market research highlighted that the current global market size of prepregs is forecast to reach USD 25.67 billion by 2031 [58]. The report further emphasized that there could be a rapid growth in carbon fiber prepregs, which is currently dominating the current prepreg market representing over 84% of the total market value. This prediction is based on the fact that automotive manufacturers are forced to utilize carbon fiber composites and reduce the overall weight of automobiles, which helps to reduce carbon emissions and increase fuel efficiency [58]. A market report published by Lin highlighted the gradually increasing demand for carbon fiber reinforced polymer (CFRP) composites from 2008 to 2025 [1,59]. It is predicted that the CFRP demand will be increased to 285 kilotons in 2025. The prepreg layup, pultrusion, and winding processes represent over 50% of the total global CFRP demand by process (Figure 5a). When compared to other composite manufacturing processes, the pultrusion and winding process has the least material waste, thus increasing the demand for CFRP production [1].

Meredith et al. highlighted that the cost of CFRP per kilogram remains at £6.38 for many industry sectors, which is much higher than the steel (£0.30/kg) and aluminum (£1.36/kg) sectors [60]. Moreover, the requirement of a high energy demand for the operation of autoclaves, the maintenance of clean rooms, and highly skilled labor have made prepreg fabrication and processing more expensive than those of conventional composite manufacturing methods starting from dry fiber and resin. Market research conducted by IndustryArc emphasized that the prepreg market is mainly governed by technology launches, acquisitions, and research and development (R&D) activities [61]. Because of these limitations, along with massive R&D activities and the limited availability of resin formulations, the global prepreg industry has been confined to several giant composite manufacturers as seen in Figure 5b.

## 3. Resin Matrix for Epoxy Prepreg Manufacturing

The resin is one of the main components of prepregs, usually representing 31% to 42% of the total prepreg composition [62]. The main function of the resin matrix is to act as the medium for the reinforcement material while providing consistency to the composite material. The resin in a prepreg usually remains in B-stage or partially cured stage until it is used in the final application. During the post-curing, the resin material undergoes a chemical reaction resulting in a hardened composite material with improved mechanical and physical properties. In this section, the important resin parameters for prepreg manufacturing, including B-staging, viscosity, and tackiness, are discussed.

### 3.1. Key Parameters for Epoxy Resin Matrix Development

#### 3.1.1. B-Stage Control

**Figure 6:**
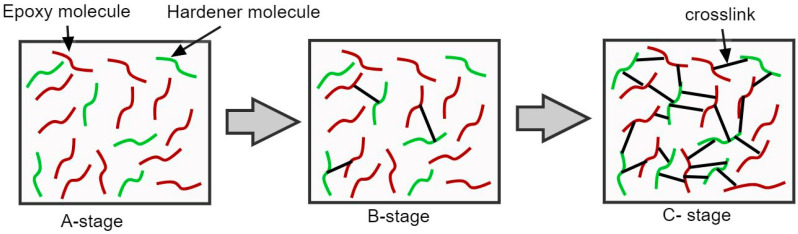
Illustration of different stages in a thermoset resin based on the number of crosslinks.

Most of the commercially available prepregs are made with thermosetting resins such as epoxies. However, there can be thermoplastic prepregs and hybrid-type prepregs for specific applications. The thermoset resins can be crosslinked using various types of curing agents. The crosslink density in a thermoset resin can be determined using the degree of cure, based on which any thermoset composite can be categorized into three major stages: A-stage, B-stage, and C-stage (Figure 6). The A-stage refers to the initial stage when the epoxy and curing agent molecules exist as separate components with zero crosslinks, whereas the C-stage refers to the resin system with a high cross-link density. The C-stage is achieved by the post-curing process [63]. The B-stage occurs in between the A- and C-stages where the thermoset is partially cured with fewer crosslinks so that the resin viscosity can still be controlled by temperature [64]. Most of the commercially available epoxy resin matrices are in the B-stage condition. In general, the B-stage resins do not cure at room temperature and can be stored as solid composites for longer periods [64]. However, the shelf life of the prepreg material is highly dependent on the type of curing agent in the matrix, and, hence, further cross-linking could occur if the matrix contains a low temperature curing agent, especially during transportation and storage.

The control of the B-stage or degree of cure is vital when developing epoxy prepregs as it accounts for viscosity and tack; two other important parameters for prepreg resin development. Further, the optimum B-stage is crucial for maintaining the drape, tack, and optimum handling of the prepreg at different temperatures. If the degree of cure is too low, it may result in excellent handling and drape properties, but it could lead to insufficient tack. On the other hand, a high level of degree of cure may result in the poor handling of the prepreg [65]. There are different approaches to controlling the B-stage and the most common method is to expose the resin mixture for a specific time at room temperature. In addition, some studies have used an elevated temperature while some have used different types of hardeners to control the B-stage [65,66]. Table 3 summarizes some of the recent studies with different B-stage control strategies.

Mohan et.al. developed an in-house unidirectional carbon/epoxy prepreg for aerospace applications by modifying an existing prepreg manufacturing process [37]. The authors employed methyl tetrahydrophthalic anhydride (MTHPA) as the curing agent to cure the resin. However, the drapability, which solely depends on the viscosity of the resin, was visually inspected. This is a major drawback of this study as the control of viscosity plays a major role in the epoxy prepreg manufacturing process.

**Figure 7:**
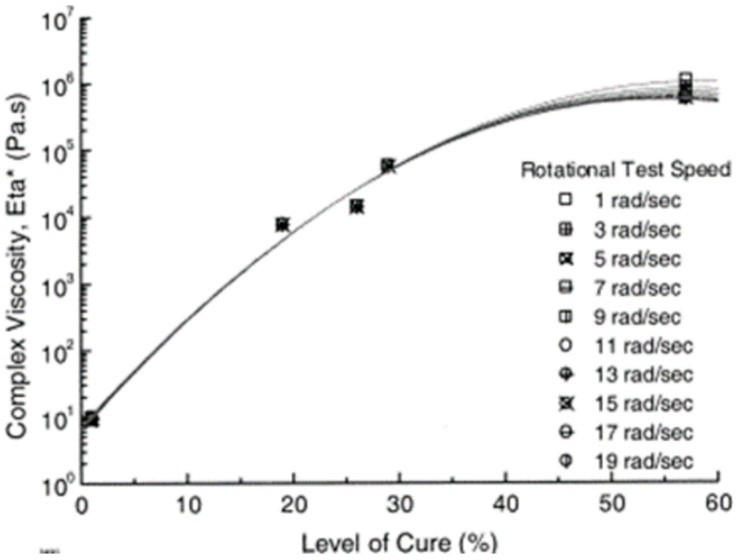
Level of cure vs complex viscosity of the prepreg. Reproduced with permission [65]. Copyright © 2004 Elsevier.

Banks et al. conducted a case study on the development of a glass/epoxy prepreg for marine and civil infrastructure applications [65]. The degree of cure was varied by holding the prepreg at room temperature for different times in order to determine the optimum degree of cure for better handling, drape, and tack properties of the developed prepreg. The use of the term ’rotation speed’ instead of oscillation could be contradictory as the use of rotation could possibly lead to breaking the crosslinks in the resin matrix. As per the results, the complex viscosity is increased when increasing the degree of cure. Furthermore, it is interesting to note the typical Newtonian behavior of the resin mixture with the degree of cure below 30% as the viscosity is independent of the rotational frequency. However, when the degree of cure is further increased from 30% to 57%, the viscosity changes with the rotational speed, revealing the non-Newtonian behavior of the resin matrix (Figure 7). The overall results suggested that 30% of cure could be the best for prepreg properties with adequate resin viscosity.

Recently, several researchers attempted to manufacture different types of prepregs by using different resin formulations and B-staging conditions [4,67,68]. Surprisingly, all these studies lack information as to how the B-stage was determined. Two types of natural fibers were used to produce epoxy prepregs by Dalla Libera Junior et al., in two different studies [67,68]. However, the effect of fibers on B-staging has not been discussed or analyzed. Similarly, Karakurt et al. studied the effect of the poly(amidoamine)(PAMAM) functionalized graphene oxide on the electrical and thermal properties of carbon/epoxy prepregs [4]. The effect of the addition of graphene oxide on the degree of cure and the viscosity of the resin matrix were not determined. Several studies have shown that the addition of particles could enhance the viscosity, thereby affecting the B-stage conditions [69,70,71]. Pouladvand et al. presented a different approach to controlling the degree of cure by changing the amount of the low-temperature curing agent along with the temperature [66]. Interestingly, they used off-stoichiometric levels of the low-temperature hardener and presented the linear relationship between the amount of hardener and degree of cure. The study suggested that the approach can be used to produce prepregs with a tailored tack and drape. Budelmann et al. evaluated the effect of B-staging on the prepreg tack [69]. The cure enthalpy values obtained from the DSC analysis were used to calculate the B-stage using Equation (1):

(1) $$\alpha =1-\frac{H_{res}}{H_{R}}$$

where α is the degree of cure, $H_{res}$ is the residual heat enthalpy, and $H_{R}$ is the total reaction enthalpy [69]. (More details about this study can be found under Section 3.1.2.)

It is important to notice that most of the studies discussed above have used low-temperature curing agents, and, hence, the control of B-state is crucial. However, prepreg production through a hot-melt resin impregnation approach can be used to avoid the drawbacks of B-stage control and is discussed in later sections in this review.

#### 3.1.2. Viscosity and Flow

The viscosity and flow of the resin matrix are some of the key factors to consider when manufacturing prepregs as they directly affect the resin impregnation, drape, tack, and final properties of prepregs [29,65,69,72,73,74,75]. Flow viscosity is important for determining the viscosity of uncured resins, whereas complex viscosity plays a major role in setting up the resin impregnation temperature and pre- or post-curing cycles of prepregs [65,74]. The resin matrix viscosity of prepregs is highly dependent on a few factors including the type of resins, impregnation temperature, curing agents, and tougheners used in prepregs [74,76]. According to Theriault et al., some of the processing conditions, especially in thermoset prepregs, including applied pressure and curing temperature profiles, solely rely on the minimum viscosity and gelation point [76]. There is a high chance of obtaining improper fiber wetting, resulting in very low interactions between the resin and fiber if the minimum viscosity is very high. At the same time, the low-viscosity resin could lead to resin bleed, resulting in imperfections throughout the composite sheets [77]. Zu et al. further highlighted that maintaining the viscosity is crucial during the Automated Fiber Placement (AFP) as an improper resin viscosity may result in slippage and bridging during the fiber layup process [76].

A perfect combination of the resin viscosity and flow is important in order to control the defects in prepregs in addition to the layup process. A mini-review focused on the manufacturing defects of complex-shape prepreg-based composites discussed that resin distribution is one of the key factors that lead to layup defects during the manufacturing of prepregs [28]. The study further highlights that a low resin viscosity could cause inter-laminar defects. For instance, the work has shown that the low viscous resin could cause more resin loss during the complex shape manufacturing processes. The study further concludes that the resin viscosity is the main factor to influence the percolation flow.

*Effect of Type of Resin and Their Composition on Resin Viscosity*

There is a wide range of epoxy resins and, mainly, the bisphenol-A epoxy resin is commonly used to produce prepregs for most commercial applications. Most of the published research on prepreg resin development have used bisphenol-A epoxy resins in liquid states [37,65,66,67,78]. In this case, the viscosity of the matrix is controlled by the curing agent itself. However, Kim et al. have used a different approach to control viscosity by employing a solid bisphenol-A resin along with a liquid bisphenol-A resin, and to make the final resin mixture to optimize the viscosity and tackiness of their epoxy prepreg [74]. The patent published by Honda et al. highlighted the epoxy resin composition for carbon-fiber prepregs with superior flame retardance and mechanical properties that can be used to manufacture electrical and electronic equipment [79]. In this patent, the authors have recommended both the upper and lower limits of viscosity of resin that are essential to maintain during the resin impregnation process. Herein, they highlight that, at 60 °C, the resin viscosity has to be between the 10 to 700 Pa.s range. They further claimed that, if the viscosity is below 10 Pa.s at 60 °C, the resin would flow down to the bottom fiber layers resulting in a low tack on the surface. Further, this may increase the resin flow during molding, resulting in irregular surfaces in the final product. If the resin viscosity is beyond 700 Pa.s at 60 °C, it may be difficult to impregnate the resin into fiber resulting in prepregs with patchy surfaces. This may further affect the moldability of prepregs.

**Figure 8:**
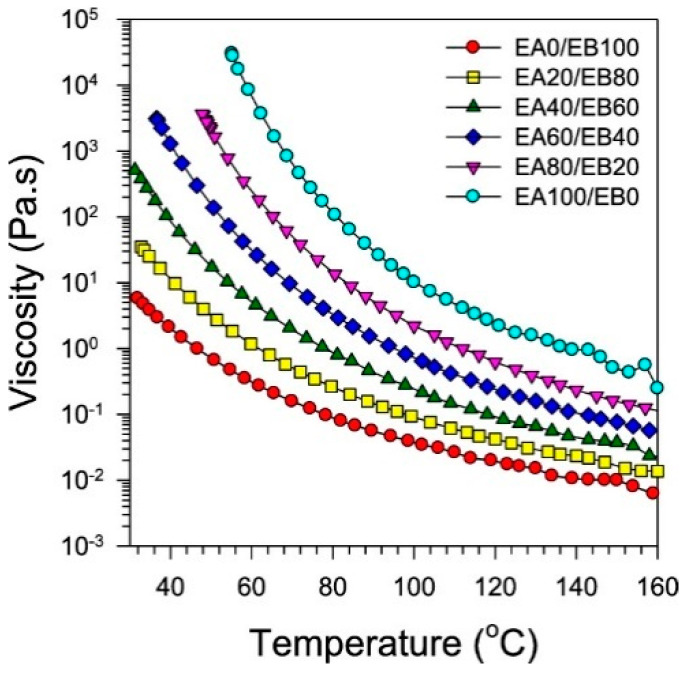
The viscosity of epoxy resin mixture with different resin ratios (EA and EB denote the solid and liquid resins, respectively). Reproduced with permission [74]. Copyright © 2021 American Chemical Society.

A recent study published by Kim et al. also highlighted the importance of maintaining the viscosity when manufacturing prepregs [74]. They have mixed different ratios of two types of diglycidyl ether of bisphenol-A (DGEBA)-based epoxy resins (having two epoxy equivalent weights) to bring the viscosity of the resin mixture to the range between 10 to 700 Pa.s at 60 °C as highlighted in the patent published by Honda et al. [79]. They used a mixture of solid and liquid types of resin and measured the viscosity of mixtures with different resin ratios with respect to the temperature in order to determine the best resin ratio (Figure 8) [74].

Based on the recommended viscosity at 60 °C, the authors have selected EA60/EB40 as the best resin ratio for prepreg development [74]. Although the recommended viscosity has been achieved through an epoxy mixture, the reason for using solid- and liquid-state resins is still unclear. In general, the solid resins could provide more tackiness and strength due to their high epoxy equivalent weights (EEWs) while the liquid resin with lower EEWs could enhance the flow properties, which are necessary for the better impregnation of resin into the fiber [80,81,82]. Further, liquid resins are more suitable for B-stage curing [80]. This could be the reason for the usage of solid and liquid DGEBA-based epoxy resin mixture.

*The Effect of Curing Agent (Hardener) on Resin Viscosity*

The use of curing agents or hardeners has been the most common practice for controlling the resin viscosity in epoxy-based prepregs. Few studies on epoxy prepreg development have employed hardeners to control the viscosity and tack of the resin during the resin impregnation process [65,66]. In these studies, curing agents have been used to control the degree of cure and the complex viscosity has been measured. However, as discussed above, Honda et al. and Kim et al. have used only latent curing agents, which do not affect the degree of cure during the resin impregnation process [74,79]. As such, they have controlled the viscosity and tack of the resin by employing a resin mixture with different EEWs as highlighted in the previous section. This is an important strategy for suppressing the crosslink reaction and enhancing the storage life of prepregs. More details on latent curing agents for prepregs and the hot-melt process are discussed in later sections.

In an attempt to develop a novel custom-tailored epoxy prepreg system, the researchers have employed diethylenetriamine (DETA) as the low-temperature curing agent to control the viscosity of resin soon after the impregnation process [66]. Therein, an off-stoichiometry ratio of DETA to DGEBA has been used to precure the prepreg along with dicyandiamide (DICY) as the latent curing agent and 1,1-dimethyl, 3-(3′,4′-dichlorophenyl) urea (commonly known as Diuron) as the accelerator. The chemical structures and physical properties of these three substances are shown in Table 4.

When determining the B-stage of resin, it is important to notice the linear relationship between the degree of cure and the amount of curing agent due to the polyaddition curing reaction between DGEBA and DETA. The degree of this reaction mainly depends on the availability of functional groups. Therefore, by limiting the amount of the curing agent, the degree of cure can be suppressed. The researchers have predicted the degree of cure with respect to the amount of DETA and have confirmed the results by the Differential Scanning Calorimetry (DSC) test. Although the authors have tried to relate the viscosity of resin by conducting probe tack and drape tests, the resin viscosity has not been determined in the study [66].

*The Effect of Temperature on Resin Viscosity in Prepregs*

**Figure 9:**
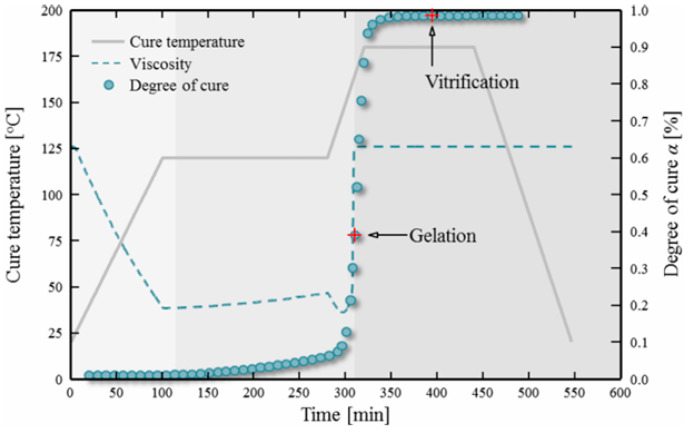
A typical curve showing the change in viscosity and degree of cure with respect to time and temperature in a multi-step cure cycle of a prepreg. Reproduced with permission [83]. Copyright © 2018 Springer-Verlag London Ltd., part of Springer Nature.

Temperature is one of the key factors that control the viscosity and flow of resin and plays a major role in the epoxy resin impregnation process. Moreover, the control of the B-stage is often carried out by changing the resin temperature (Table 3). A general curve that shows the changes in resin viscosity and degree of cure with respect to the temperature (curing cycle) of an epoxy prepreg is shown in Figure 9 [83]. At the beginning, the viscosity drops with increasing temperature, and, once the temperature reaches the curing temperature, the epoxy starts developing chemical crosslinks. This results in a rapid increase in viscosity, followed by a plateau indicating the maximum number of crosslinks in the epoxy resin.

**Figure 10:**
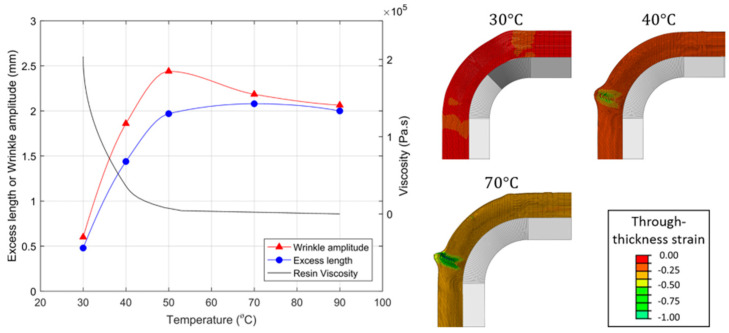
Effect of temperature on viscosity and wrinkle severity. Reproduced with permission [84]. Copyright © 2018 The American Society of Mechanical Engineers.

Belnoue et al. highlighted the effect of the viscosity of resin on the fiber waviness using predictive numerical models [84]. Accordingly, the authors have used temperature as the parameter to vary the viscosity and summarized the correlation between the resin viscosity and the wrinkle severity as depicted in Figure 10. The results showed a clear increase in excess length and wrinkle amplitude at lower viscosity levels. As such, the control of temperature is very important for controlling the defects in prepregs, especially due to resin viscosity variations.

**Figure 11:**
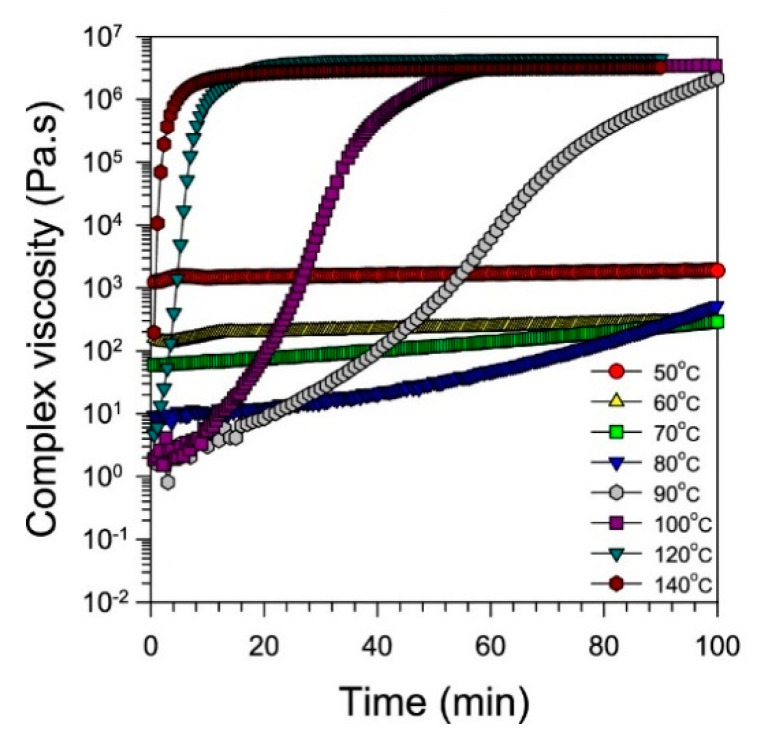
Complex viscosity of resin matrix under isothermal conditions. Edited and reproduced with permission [74]. Copyright © 2021 American Chemical Society.

Kim et al. employed the hot-melt impregnation technique to impregnate resin into carbon fibers [74]. Therein, the viscosity of the resin is reduced by increasing the temperature to achieve better wettability and complete impregnation during the impregnation process. In order to determine the optimum impregnation temperature, the complex viscosity of the resin matrix is measured at different isothermal curing temperatures between 50 to 140 °C on a rheometer (Figure 11). The outcome showed that the temperatures lower than 70 °C had no significant increase in viscosity (or curing), and, hence, the optimum resin impregnation temperature is set between 60–70 °C.

Few studies have indicated that viscosity and temperature are two major parameters that affect interplay friction, which leads to the formation of a wrinkle effect through the ply slippage in complex-shaped prepregs [71,85,86,87]. Wang highlighted the correlation between the temperature-dependent viscosity of the resin matrix and dynamic friction through Equation (2), where τ is the shear stress, γ is the shear rate, $\eta \left(T\right)$ is the temperature-dependent resin viscosity, h is the thickness of the viscous fluid layer, and ν is the lateral velocity [71]:

(2) $$\tau =\gamma \eta \left(T\right)=\frac{\nu }{h}\eta (T)$$

As indicated by Equation (1), the processing temperature can significantly affect the dynamic friction among prepreg plies as it accounts for controlling the viscosity and flow during the post-curing stage.

*The Effect of Tougheners on Resin Viscosity in Prepregs*

Tougheners are often used along with epoxy resins to improve the fracture toughness and improve the damping properties in high-performance aerospace-grade prepregs [27,88,89]. The commonly used tougheners in epoxy resins are high-molecular-weight thermoplastics including polyethersulfones (PESs) and polyimides (PEIs) owing to their ability of forming two-phase morphology in the epoxy resin matrix, thus improving the crack propagation [69,70,71,90,91,92,93]. In addition, several reviews have been conducted on a wide range of toughening materials including core-shell rubber particles, liquid rubbers, dendritic polymers, block copolymers, rigid particles, and soluble thermoplastic fibers [88,89]. Although the use of tougheners brings important benefits, their effect on resin viscosity and tack could be problematic, especially during the resin impregnation, prepreg layup, and post-curing [89]. Budelmann et al. incorporated PESs into epoxy prepolymer, and tetrafunctional tetraglycidyl-4,4′-methylenedianiline (TGMDA) by mixing PES powder with preheated resin [69]. The results showed the rise in the complex viscosity and glass transition temperature with an increase in PES content from 10 to 30%. The authors claimed that the system with a 10% PES content and the B-stage level of 20% showed similar values for a commercially available aerospace prepreg in terms of their tack. Galledari et al. fabricated a solid acrylonitrile–butadiene rubber (NBR) toughened epoxy/glass prepreg by the hot-melt method [94]. The amount of NBR increased from 0 to 5% and the rheological results indicated an increasing trend of both gel times due to the increasing viscosity.

**Figure 12:**
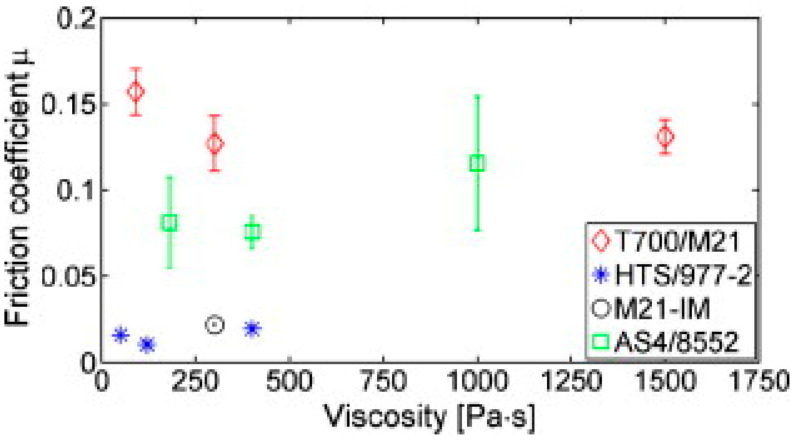
Friction coefficient at different viscosities. Reproduced with permission [87]. Copyright © 2011 Elsevier.

Having difficulty in extracting the resin from commercially available prepregs (due to the B-stage condition of the prepreg resin matrix) and mixing tougheners, several researchers have fabricated the tougheners on the prepreg surface to make interlaminar toughened composites [27,71,95,96]. However, the main drawback of this process is the limited use of toughener amounts owing to the increase in resin viscosity and enhanced particle agglomerations [27,71]. Laberg et al. evaluated the interplay friction force of four unidirectional carbon/epoxy prepregs, one (T700/M21) having a resin layer with toughened particles [87]. The friction coefficient force was calculated at similar viscosity ranges, which resulted from different temperature ranges. The results indicated a significant increase in the friction coefficient of the prepreg with toughened particles compared to the other three prepregs as shown in Figure 12.

The above-discussed studies clearly outline that the type and the quantity of tougheners are some of the key factors that affect the final viscosity, gel time, and tack of resin matrix in prepregs.

*Resin Viscosity Measuring Techniques*

**Figure 13:**
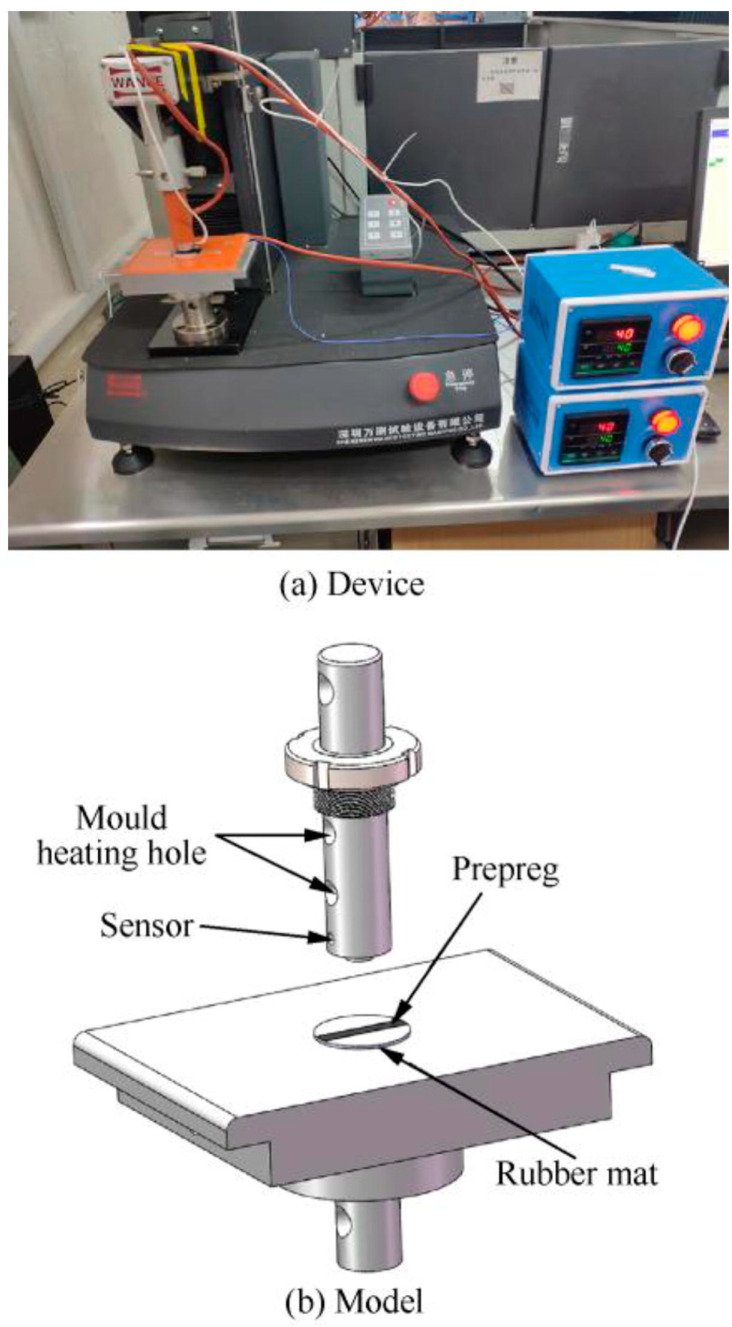
(**a**) Probe test device (Shenzhen Wance single-column mechanical testing machine) and (**b**) model. Reproduced with permission [99]. Copyright © 2022 Elsevier.

Researchers have employed different techniques to determine the viscosity of resin in prepregs. The quantitative analysis of prepreg viscosities by probe, peel, and tension or compression tests is highlighted in a few studies [29,65,66,97,98,99]. Among these three methods, the probe and tension/compression methods follow the same principle as they use a load separation process to determine the viscosity of prepregs [99]. During the probe test, the prepreg surface is pressurized with a defined force by a probe head for a certain period, and, subsequently, the probe head is released at a constant rate while measuring the load displacement force. The probe test device and the model are depicted in Figure 13a,b.

The probe test is also used to measure the tack of prepregs, which will be discussed in a later section of this review. It is interesting to note that the peel method provides more reliable results as the tests are conducted through a laid prepreg. Moreover, the peel method provides more benefits over the other methods owing to its ability to measure the bending stiffness and adhesive forces between prepreg layers [100,101,102]. By considering these benefits, Zu et.al. compared the probe and peel tests using a commercially available prepreg [99]. A peel test device was employed to measure the load displacement during the peeling process. Subsequently, the authors established a relationship between these two tests through a peel simulation study.

**Figure 14:**
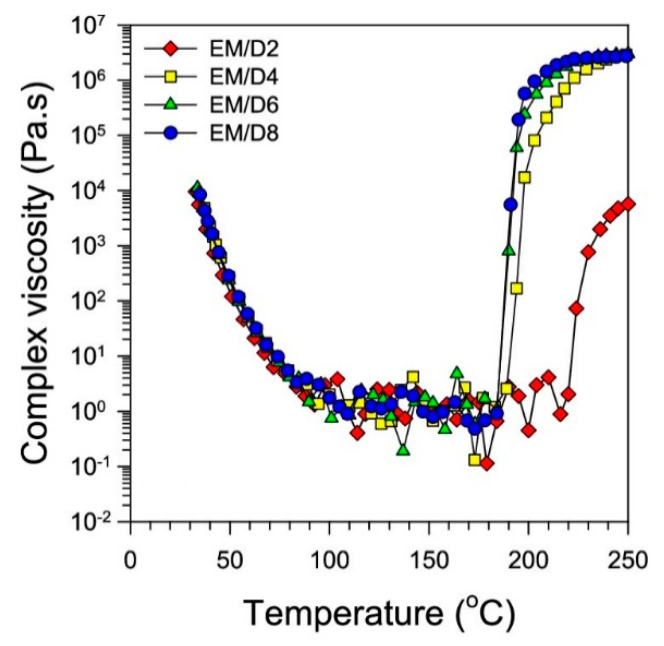
Complex viscosities of epoxy resins with different curing agent (DICY) loadings Reproduced with permission [74]. Copyright © 2021 American Chemical Society.

Kim et al. utilized a parallel plate rheometer to determine the viscosity of epoxy resin [74]. Therein, the steady shear viscosity of neat epoxy as a function of temperature and at different shear rates was determined to assess the flowability of epoxy resin mixtures. The dynamic rheological properties and complex viscosities of epoxy resin mixtures with curing agents were determined by the oscillatory shear mode in the rheometer (Figure 14). Complex viscosity studies are extremely important for highlighting the curing behavior of prepregs.

Banks et al. measured the complex viscosity of prepreg at different levels of cure using a parallel plate rheometer [65]. They further investigated the effect of oscillating frequency on the complex viscosity of epoxy-based prepreg. The surface friction is considered a crucial factor for prepreg layup and is mainly determined by the viscosity of the partially cured resin in the prepregs [71]. As such, some researchers employed a parallel plate rheometer to determine the prepreg surface friction considering its ability to control the temperature and force precisely [71,103]. However, this technique measures the rotational friction force which resulted from the variable rotation speed and fiber orientation and, therefore, cannot be separately analyzed from the effect of temperature. This is considered a critical shortcoming of the use of a rheometer for surface friction analysis [71].

#### 3.1.3. Tackiness of Prepregs

The tackiness of a prepreg is another important factor that can be mainly achieved by the careful selection of the resin matrix. The tackiness or stickiness is important, especially during the layup process of uncured prepregs [28]. The right amount of tack of a prepreg would lead to easy handling and excellent laminate properties. The lack of proper tack properties could lead to bonding defects during the layup process including bridging and wrinkling, and, ultimately, a material loss [29]. Prepreg tack is mainly governed by adhesive and cohesive interactions and is not attributed to any chemical reaction taking place during the curing of prepregs [29]. The aforementioned intrinsic interactions are greatly affected by the viscosity of resins, processing parameters, and environmental factors [29,99].

A recent review published by Budelmann et.al. on the effect of prepreg tack during the automated layup processes discussed the factors affecting prepreg tack in detail [29]. As per the review, the influential factors are classified into two categories: environmental aspects during production (extrinsic) and prepreg material properties (intrinsic). The intrinsic factors are more important for controlling tack during the early stages of prepreg development, whereas the extrinsic factors are important during the layup or molding process. Table 5 summarizes the intrinsic properties that affect the prepreg tack.

**Figure 15:**
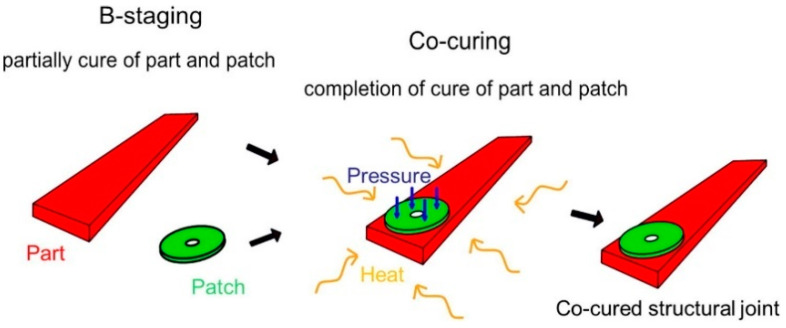
Concept of co-curing and joining of B-stage components using resin tack. Reproduced with permission [104]. Copyright © 2016 Elsevier.

The intrinsic parameters are extremely important for developing tailor-made prepreg systems and can be mainly characterized by rheological, thermal, and microscopic analysis during the early stages of prepreg development. Studer et.al. demonstrated the use of tackiness to join B-stage carbon/epoxy composites without an adhesive layer [104]. The study uses a kinetic model to describe the co-curing of B-stage components as shown in Figure 15 and, finally, highlights the possibility of using this technique for combined manufacturing processes such as resin infusion, prepregs, conventional resin transfer molding, or compression resin transfer molding.

**Figure 16:**
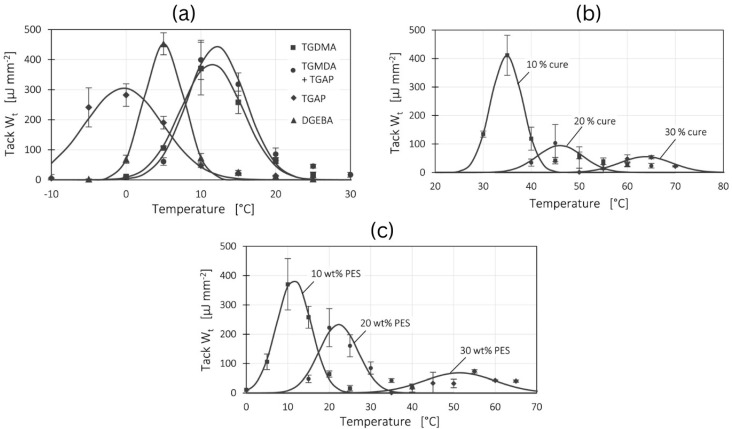
The effect of (**a**) prepolymer, (**b**) B-staging, and (**c**) toughener content on prepreg tack. Edited and reproduced with permission [69]. Copyright © 2022 Elsevier.

Banks et al. highlight the reduction in tack properties with a decreasing degree of cure [65]. (See Section 3.1.1 for more detailed discussion.) The effect of resin formulation, B-staging, and toughening on epoxy resin tack has been recently studied by Budelmann et al. [69]. The study used different epoxy prepolymers including tetrafunctional tetraglycidyl-4,4′-methylenedianiline (TGMDA), a trifunctional triglycidyl p-aminophenol (TGAP), and a bifunctional Bisphenol A diglycidyl ether (DGEBA) to highlight the effect of the prepolymer on the resin tack properties (Figure 16a). Based on previously published results by the same researchers, it was concluded that the tack of all A-stage prepolymers is 3 to 4 times higher than that of commercial prepregs [73,105]. The degree of cure results indicated that the tack bell curves were moved towards higher temperature regions with decreasing tack, which may be attributed to the reduction in resin viscosity at higher temperatures (Figure 16b). Moreover, the tack was reduced by increasing the toughener (high-molecular polyethersulfone) content as shown in Figure 16c.

*Tack Measurement Techniques*

Until the introduction of the standard test method for the characterization tack of prepregs by ASTM D8336 in 2021, there was no standard method to test the tack in prepregs [106,107]. Prepreg developers often used trial-and-error methods or previous knowledge rather than proper technical data. The information provided by commercial suppliers on the prepreg tack was very little, which could be a possibility due to the large number of influential factors on the tack. Most of the available tack-measuring techniques were linked to pressure-sensitive adhesive (PSA) technology. Due to the adhesive nature of prepregs, scientists have tried to use similar PSA standard tack-measuring techniques, namely, the probe tack test and peel test, to determine the tack of prepregs [29,65,98,102]. However, the recently introduced ASTM standard test for the characterization of prepreg tack uses the peel test method and is discussed in detail below [107].

- *Probe Tack Test*

The probe tack test is often used by researchers owing to its excellent control of input variables and high precision when compared to other available tests. It is generally performed on a universal testing machine with a special fixture mounted on it [29,66]. Recently, several researchers have performed tack measurements using a rheometer as it allows for controlling the temperature and measuring relatively low forces with great accuracy [73,108,109,110].

**Figure 17:**
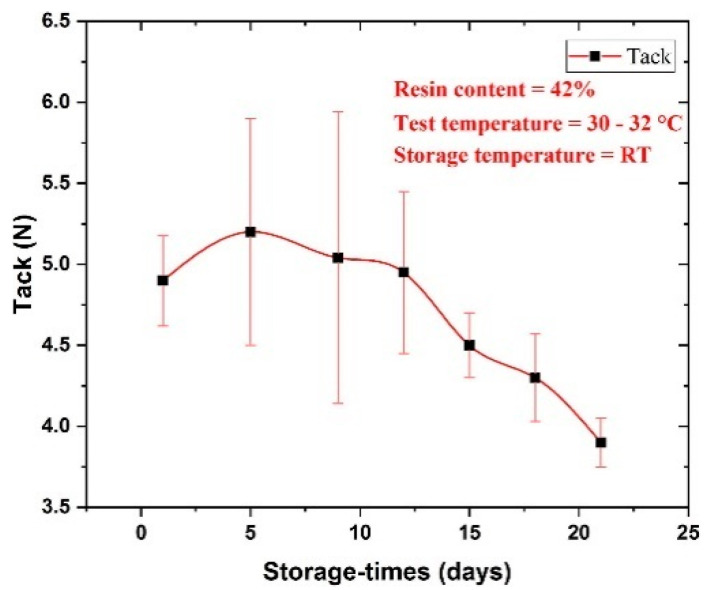
Illustration of probe tack test and the outcome. Reproduced with permission [66]. Copyright © 2020 Elsevier.

This test has two separate phases: the compression phase and the tensile phase. During the compression phase, a downward pre-defined force is applied on the prepreg through a flat-head probe for a definite time. Afterward, the pressure probe is moved upward during the tensile phase with a defined separation rate, and the maximum resistive force against this motion is recorded as a negative force value [29,66,99]. Pouladvand et al. have illustrated the process steps of the probe test and force variation, and are shown in Figure 17 [66]. The study proposed the probe test as an alternative simple test that can replace complex and expensive tests available for evaluating the prepreg quality.

**Figure 18:**
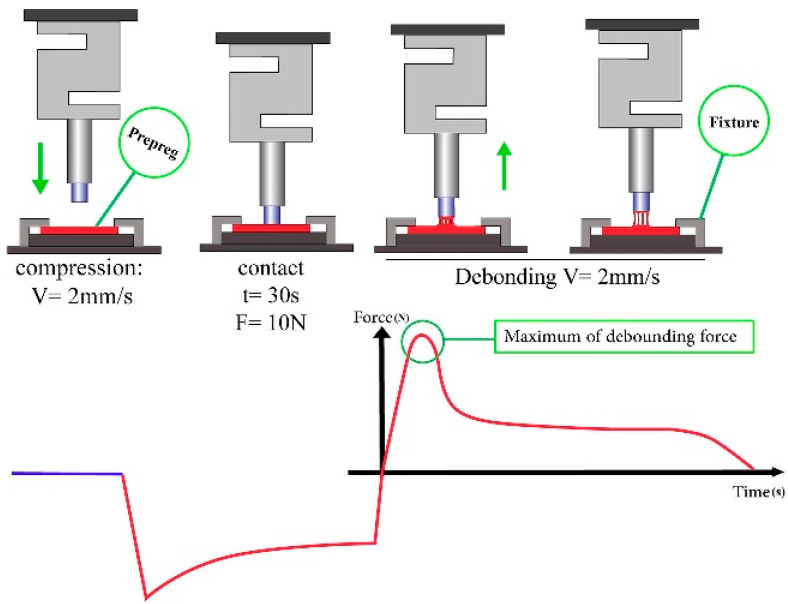
The measurement of tack of a dual-curable prepreg over a period. Reproduced with permission [66]. Copyright © 2020 Elsevier.

The change in tack with the storage time of 25 days under room temperature is revealed in Figure 18 [66]. Based on the study results, the tack increases for the first five days of storage time, which may be ascribed to the incomplete pre-curing in the presence of an aliphatic amine agent. Subsequently, the tack reduces with increasing storage time as the resin starts to flow in a B-stage prepreg [66]. This is a good indication that prepreg reduces its quality over time if it is stored under normal temperature.

**Figure 19:**
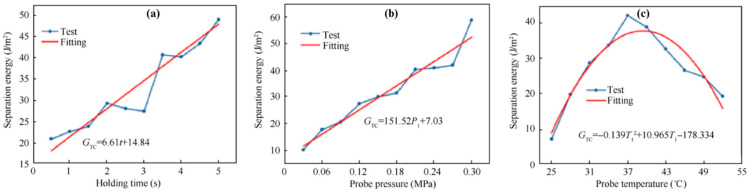
Effect of (**a**) holding time, (**b**) probe pressure, and (**c**) probe temperature on the separation energy. Reproduced with permission [99]. Copyright © 2022 Elsevier.

A study conducted by Zu et al. investigated tack properties of Toray’s T700-HX7901 using the Shenzhen Wance single-column mechanical testing machine (Figure 13) [99]. The researchers have used the same material (stainless steel 304) as the pressure roller for the gasket to meet the realistic conditions of the prepreg fabrication process, and, at the same time, have employed a temperature control box along with mold heating tubes to heat the probe and the mold from 25 to 52 °C. Rather than reporting the direct tack in terms of the separation force, the authors have tried to correlate the tested parameters of the probe test, which includes the holding time, probe pressure, and probe temperature, with the prepreg resin viscosity. The study concluded that the viscosity of the prepreg increases when increasing the probe holding time and the probe pressure within a certain range (Figure 19a,b), whereas, with increasing temperature, the viscosity first increases and then decreases with a peak separation energy around 37 °C (Figure 19c).

b. *Peel Tests*

**Figure 20:**
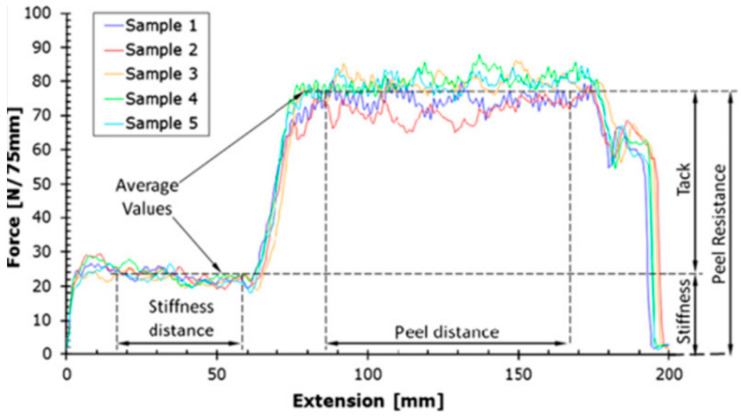
Peel test output showing stiffness and tack. Reproduced with permission [98]. Copyright © 2012 Elsevier.

In the peel test, mainly the peel angle (90°, 180°, or T-Peel) and type of testing equipment have been considered when developing testing standards for the peel tests in PSA tapes [29]. During the general peel test, the tested material is removed from a defined substrate or itself while maintaining a constant peel angle. The same principle has been used to measure the tack of prepregs with some modifications. The tack is evaluated in terms of the average load and work of adhesion with respect to the measurement distance or displacement [29,98]. Crossley et al. have suggested an extension to an existing British standard peel test by including a pressure control application stage to measure the dynamic stiffness of the uncured prepreg in addition to the tack (Figure 20) [98]. Therein, the authors have developed a setup that could perform prepreg laying and peeling at the same time. Although they have obtained consistent results with a 16% standard deviation, the actual laying rate could not match the experimental laying rate. The study further suggested that not only resin but also the fiber surface and impregnation effects should also be considered when specifying the tack of prepregs.

**Figure 21:**
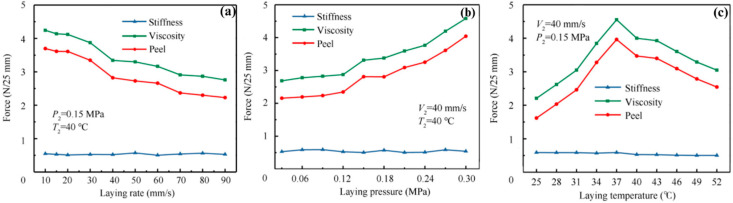
Peel test results under different (**a**) laying rates, (**b**) laying pressure, and (**c**) laying temperature. Reproduced with permission [99]. Copyright © 2022 Elsevier.

Zu et al. have employed both the peel (90°) and probe tests to characterize the viscosity of prepregs in terms of peel force [99]. The overall study results showed that the viscosity of prepregs is proportional to the laying pressure, inversely proportional to the laying rate, and quadratic to the laying temperature. In this study, different laying conditions have been used in terms of the laying rate, pressure, and temperature and the results are shown in Figure 21. The results showed that the prepreg viscosity decreases with an increasing laying rate, whereas it increases with decreasing pressure. However, when increasing the temperatures, the viscosity increases first and then decreases after 37 °C.

**Figure 22:**
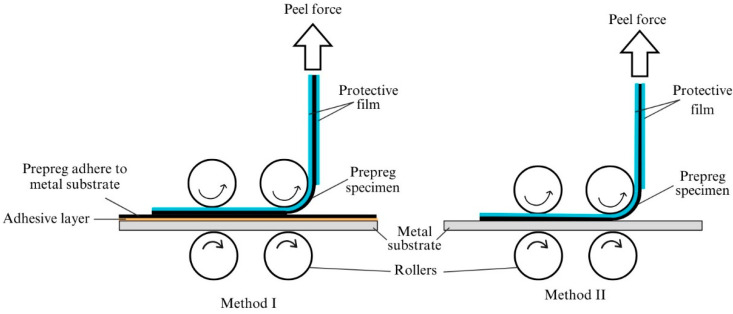
Schematic diagram showing two methods of continuous application-and-peel test mentioned under ASTM D8336 [107].

Bank et al. have selected ASTM D3167 to measure the tack of their prepregs in different cure levels [65]. The standard was originally developed to determine the floating roller peel resistance of pressure-sensitive adhesives [111]. According to the standard, one adherent has to be rigid, and the other adherent has to be flexible. A load is applied through the flexible adherent at a pre-defined separation rate over a specific length of the bond line (76 mm). The ASTM D8336-24 is recently developed to quantify the tack of a prepreg at a specified condition (temperature and relative humidity) by using a continuous application-and-peel technique [106,107]. The method can be used to measure the tack between two B-stage prepreg plies (Method I) and also between the B-stage ply and rigid surface (Method II) as shown in Figure 22. When a prepreg specimen is passed through the test fixture, the compaction rollers press and bond the specimen against the substrate, and, at the same time, the prepreg is peeled off from the substrate. The peel force is measured as a function of crosshead displacement and only the peel-force-related adhesion is derived from the collected data over two different phases.

*Measurement of Tack Using a Rheometer*

**Figure 23:**
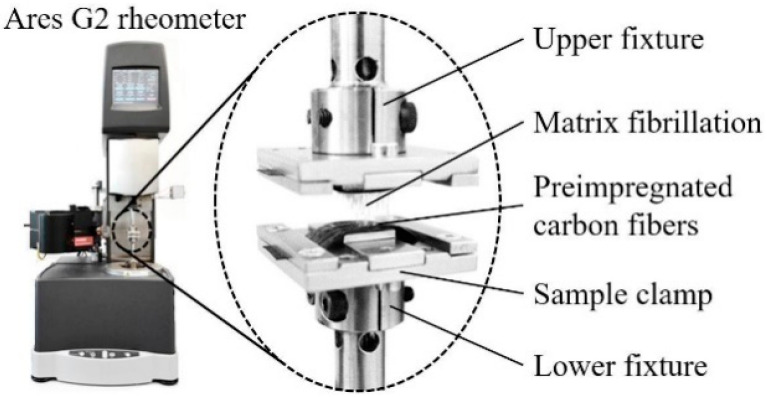
Prepreg sample holder used for tack test in the rotation rheometer. Reproduced with permission [73]. Copyright © 2019 Elsevier.

In addition to probe and peel tests, several researchers used a rheometer as a test apparatus to determine the tack of prepregs. Budelmann et al. have determined the effect of the temperature, compaction force, debonding rate, and ageing on prepreg tack with a rotational rheometer [73]. The test apparatus of the rheometer is shown in Figure 23. Although the same principles of the probe tack test have been applied for tack measurement, the authors claimed that the output is more precise owing to the ability to measure transient normal force ranging from 0.0001 to 20 N at a very high resolution of 10^−5^ N. The study concluded that the tack is greatly affected by process-related factors including temperature, layup speed, and compaction force, and material-related factors including age, matrix resin, and draping surface. The authors finally suggested an experimental validation in an automated layup process before conducting the prepreg tack adjustment on production-related aspects.

Wohl et al. have used a custom-made fixture to hold the prepreg and conducted probe tack tests of prepregs using a rheometer equipped with an environmental controller [109]. The tack was measured under different environmental conditions including temperature and relative humidity, and also several experimental configurations including contact time and crosshead speed. The study concluded, among these variables, temperature and relative humidity were the most influenced parameters while the maximum tack can be obtained under low-temperature and moderate-humidity conditions.

## 4. Important Insights in Epoxy Resin Matrix Development for Prepregs

### 4.1. Key Challenges in the Resin Formulation Development in Epoxy-Based Prepreg Development Technology

When compared to conventional composite preparation methods, the prepreg technology differs owing to the partially cured resin matrix (B-stage) available in most of the commercially available prepregs. As discussed in Section 3.1.1, control of the B-stage in epoxy-based prepreg has become one of the greatest challenges in the prepreg resin matrix development process. Further, it is hard to control the viscosity and tack independently as both these parameters are linked and can be controlled through the B-stage of the resin. Most commercial prepregs are often stored under subzero conditions to suppress further curing (storage hardening) which may reduce the tackiness and viscosity of prepregs. All these challenges equally reflect the importance of choosing a proper curing agent for epoxy-based prepreg development [65,74]. Another great challenge is the limited literature on the resin matrix development for epoxy-based prepreg development. Although there are numerous prepregs available in the market, a systematic approach to resin matrix development is barely disclosed and hidden under trade secrets. As a result, prepreg technology is scarcely linked with the most recent technologies in material science including nanotechnology and smart material development. Therefore, the establishment of a scientific approach as a base for resin matrix development for epoxy prepregs is considered a timely important task. Based on the available literature and by considering the above-discussed important factors, two important approaches for epoxy prepreg resin development are proposed and are discussed below.

### 4.2. Proposed Approaches for the Development of Epoxy Matrix Formulation

**Figure 24:**
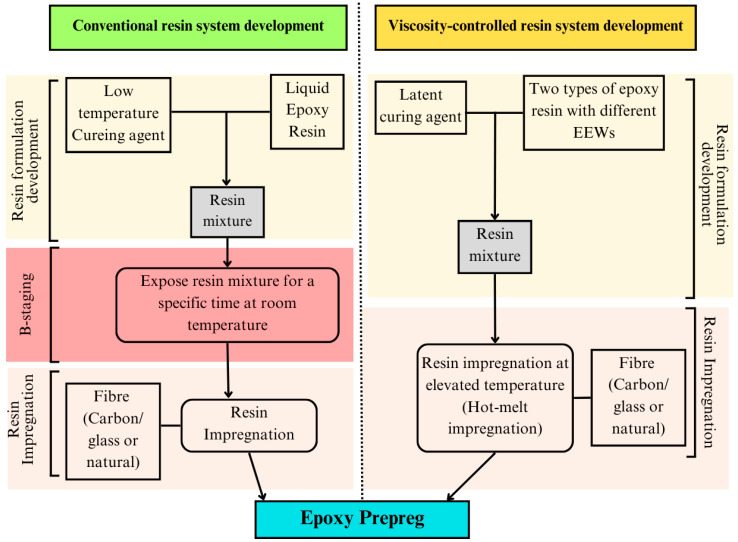
Process flow diagram showing the differences between conventional and viscosity-controlled resin systems.

Based on the resin impregnation temperature, curing agents, and resin viscosity, two approaches are proposed for resin matrix impregnation in prepregs, namely, conventional resin impregnation and viscosity-controlled resin impregnation. The main differences between these two methods are highlighted in Figure 24.

#### 4.2.1. Conventional Resin Impregnation Approach (Resin Impregnation at Room Temperature)

When developing prepregs with conventional resin systems, it is important to use a low-temperature curing agent and also to control the B-staging in the epoxy matrix. Table 3 summarizes some of the recent studies carried out on prepreg development, starting from the resin composition development, B-stating, and curing conditions [4,37,65,66,67,68]. In these studies, the degree of cure is mainly controlled by the temperature and time. Banks et al. have changed the degree of cure from 1% to 57% by exposing the prepreg for different time durations under room temperature [65]. A parallel plate rheometer was utilized to determine the degree of cure and observed the non-Newtonian behavior of the resin after 30% of the degree of cure. However, there is no indication about the determination of the pot life of the prepreg. Several studies have used room-temperature curing agents to develop epoxy-based prepregs [37,67,68]. Karakurt et al. have used the same epoxy resin but have employed elevated temperature (80 °C) conditions for B-staging [4]. Although the information on the mixing ratio of the resin and hardener is available, the study does not examine the B-staging conditions or degree of cure.

**Figure 25:**
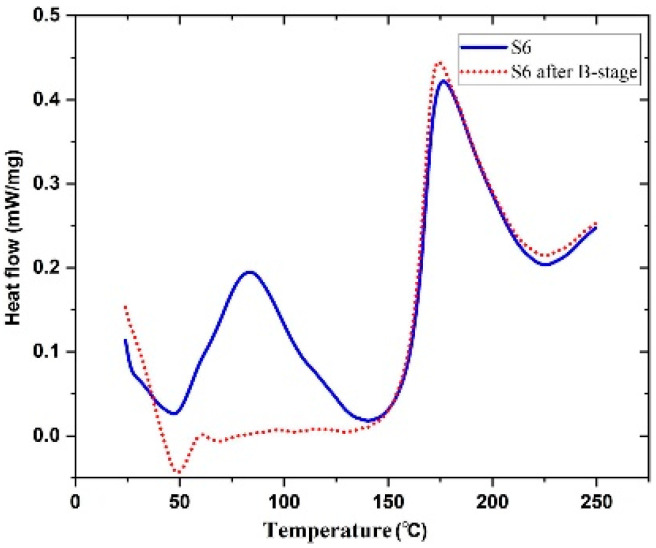
DSC diagram of before and after B-staging of prepreg with dual-curable resin system. Reproduced with permission [66]. Copyright © 2020 Elsevier.

Pouladvand et al. have used a novel approach to precisely control the degree of cure when developing prepregs [66]. Here, the researchers have employed two different types of curing agents: a latent and a room-temperature curing agent. The room-temperature amine-based curing agent (DETA) was used in off-stoichiometric ratios mainly to remove the thermal history and control the B-stage or the degree of cure of the prepreg, while the latent curing agent (DICY) was used for the post-curing of the prepreg. The DSC results before and after B-staging showed two separate curing stages (Figure 25). Based on the FTIR results of the developed prepreg after keeping it for 21 days at ambient temperature, the authors claimed that the system is capable of being stored at ambient temperatures, unlike the conventional commercial prepregs. However, they have noticed a 7% increase in the degree of cure and a clear reduction in tack prepregs (with a 42% resin content) after 21 days. These results indicated that the curing reaction cannot be stopped although they have used an off-stochiometric ratio of the low-temperature curing agent.

It is important to notice that control of B-staging has become the major challenge when developing prepregs with low-temperature curing agents as it is responsible for controlling the viscosity and tack of prepregs. Moreover, these systems do not eliminate the instability of prepregs, and, hence, should be stored under freezing conditions before use.

#### 4.2.2. The Viscosity-Controlled Resin Impregnation Approach (Hot-Melt Resin Impregnation)

*Control of Resin Viscosity*

It is understood that optimum B-staging is vital for obtaining proper tack and viscosity levels of prepregs. However, this process, in turn, results in a major drawback as most prepregs are continuously cured during storage, which ultimately lowers the shelf life, tack, and overall performance. Instead of using a low-temperature curing agent, several researchers have employed a combination of solid and liquid epoxy mixture to control the viscosity and tackiness of prepregs [74,79,82,94]. It is interesting to note that there is no B-staging or any involvement of a low-temperature curing agent in this viscosity-controlled system (Figure 24).

Honda et al. have patented an epoxy resin composition for epoxy-based carbon fiber-reinforced prepregs for electrical/ electronic equipment with excellent flame retardance and mechanical properties [79]. In their patent, the authors have used four components to develop their prepreg, namely, the resin, amine curing agent, phosphorous compound, and curing accelerator. Under the resin components, they have claimed a wide variety of epoxy resins that can be used to produce flame-retardant prepregs. Most importantly, they have recommended a range of the resin viscosity levels for prepregs as 10 to 700 Pa.s at 60 °C. Moreover, a latent amine curing agent along with a curing accelerator was utilized for curing and the optimization of the prepreg. As per the patent, the latent curing should be activated between 70 to 125 °C for low-temperature curing applications. If it is below 70 °C, it may affect the shelf life of the prepreg, while, if the temperature is well above 125 °C, the expected rapid curing may not be achieved. Two resin impregnation techniques have been mentioned under this patent. The first technique is called the wet process, in which the resin is dissolved in a solvent such as methyl ethyl ketone or methanol to reduce the viscosity, followed by impregnation into the fiber matrix. This method has been used elsewhere to develop a solid acrylonitrile–butadiene rubber (NBR) toughened epoxy/glass prepreg [94]. The hot-melt process is the second process during which the resin is heated to reduce the viscosity prior to impregnation into the fiber matrix. The hot-melt process is preferable over the wet process due to the lack of involvement of solvents. Overall, the patent gives a better insight into the development of prepregs using a viscosity-controlled resin system.

Based on the viscosity recommendation made by the above patent, Kim et al. have used a mixture of solid and liquid epoxy resins to develop their prepreg using the hot-melt resin impregnation process [74]. The study indicated a systematic determination of each component in the resin formulation (resins, latent curing agent, accelerator, and latent curing additive) through the viscosity and curing steps. The viscosity–temperature relationship has been used to determine the optimum amounts of epoxy mixture (Figure 14). Some of the details about viscosity control are already discussed in Section 3.1 in this review.

*Role of Latent Curing Agent*

One of the main differences between the two proposed approaches is the use of latent curing agents in the viscosity-controlled approach. Here, the latent curing agent does not influence the B-staging at low temperatures; hence, it can be used to develop prepregs with an extended shelf life. Honda et.al. have highlighted a wide range of curing agents including amine curing agents, aromatic polyamines, and latent curing agents [79]. The latent curing agents are often used in conjunction with curing accelerators to reduce the post-curing temperature by accelerating the curing process [74,79,94]. For example, one of the most common latent curing agents, dicyandiamide (DICY), starts its curing reaction above 170 °C. If it is used along with an accelerator (a compound that contains two or more urea bonds per molecule), it is possible to bring down both the curing temperature (between 80–150 °C) and curing time, which is more viable for industrial processes [74,79]. Another great advantage of the epoxy prepregs developed with latent curing agents is the extended shelf life. It is reported that epoxy/DICY can extend the shelf life by up to six months at room temperature [112]. This could allow such prepregs to be used for large-scale applications as there is no sudden change in B-staging or storage hardening. The lack of B-staging and storage hardening is also extremely important when it comes to resin matrix modifications and the use of different tougheners to enhance certain properties of prepregs [94].

## 5. Conclusions and Outlook

### 5.1. Conclusions

The following concluding remarks can be made based on the review output:

1. Prepregs are mainly used in four sectors: aerospace, energy, automotive, and miscellaneous (sports, smart application, etc.). Automated tape layup (ATL) and automated fiber placement (AFP) are often used for aerospace, aeronautical, and wind turbine applications. Moreover, few automobile manufacturers have already used prepregs to replace their automobile metal parts to reduce the mass and cost, especially in sports cars. Although the prepreg layup plays a major role in the CFRP market (about 25% of the total CFRP global demand by process), the global manufacturing ability is confined to very few producers. The studies on nanoengineered prepregs and smart polymer prepregs are extremely limited as most of these techniques involves modification of resin matrix which cannot easily be carried out in B-staged prepregs.
2. During the last decade, about 10 reviews have been published on prepregs and most of them focused on post-curing, prepreg layup, tack, and testing (Table 2). None of these reviews focused on resin matrix development strategies for epoxy-based prepregs. This review mainly highlighted three important resin parameters; B-staging, viscosity, and tack, and discussed how these parameters are controlled to obtain optimum prepreg resin properties.
3. B-staging, viscosity, and tack are the most important resin properties to be considered when developing epoxy prepregs. Viscosity could control the drape and resin distribution while the tack plays a huge role when fabricating prepreg laminates. Control of these properties is equally important for the B-staging and final curing of prepregs. The B-stage is mainly controlled by exposing the resin mixture to a low-temperature curing agent for a specific time at room temperature. However, few studies used temperature and different types of hardeners for the same purpose.
4. It is required that we maintain the resin viscosity in an optimum range as it directly affects the drape, tack, and fiber wetting of prepregs. The resin viscosity can be controlled mainly by changing the temperature. In addition, the type of epoxy resin and its composition, the effect of hardener, and the quantity of tougheners can also be used to control the viscosity of the resin matrix.
5. Maintaining an optimal tack is vital for better handling and prepreg layup, which helps to reduce the debonding and wrinkling defects of prepregs. The tack of a prepreg mainly depends on the resin viscosity, prepreg architecture, and degree of cure. The resin tack can be determined by the probe and peel test methods. ASTM D8336 was introduced in 2021 for prepreg tack quantification, which is based on continuous application-and-peel tests. It is found that the use of a rheometer for tack evolution is more reliable than the other techniques.

### 5.2. Outlooks

1. The most common technique of prepreg resin matrix development is the use of a room-temperature curing agent to control the degree of cure which is discussed in Section 4.2.1. Although the conventional resin impregnation approach is more straightforward, the control of the degree of cure and extension of shelf life is extremely difficult. Further, having a partially cured (B-stage) resin matrix could lead to the restriction of the further modification of the resin matrix.
2. Prepregs developed through viscosity-controlled resin impregnation approach (Section 4.2.2) do not involve any partially cured condition, thus the approach is more suitable for the development of prepregs with an extended shelf life and for large-scale applications. Very few studies have employed the viscosity-controlled resin system, where two or more epoxy resins are used to control the viscosity along with a latent curing agent for post-curing. In this approach. the lack of a B-stage is more significant and brings more benefits to the prepregs including extended shelf life, no specific storage conditions, and the possibility of modifying the resin matrix enabling the development of functional composites and smart materials.

## Figures and Tables

**Table 1:** An insight into a historical background on the development of prepregs from the late 1960s to 1991.

Year/Period	Key Activity/Event	Ref.
1960s	Early development of automated tape layup (ATL)	[16,20]
1970s	Commercial application of ATL	[16,20]
1971	A Computer Numeric Control (CNC) was developed to laminate composite tape onto a rotatable base-plate	[16]
1974	Development of an automated rotatable head for complex part manufacturing	[16,21]
1980	Initialization of the manual layup of prepregs	[16,22]
Early 1980s	Further development and improvement of ATL technique	[17,18,19]
1990s	Introduction of tape heating to overcome the defects occurred during the complex laminate layups and control the tack in large parts	[16]
1991	Introduction of irradiation heating for thermoplastic layup	[16,23]

**Table 2:** Summary of reviews published on prepregs during the last decade.

Major Focus	Review Title	Ref.
Post-curing	A review of out-of-autoclave prepregs—material properties, process phenomena, and manufacturing considerations	[13]
	A review on fabrication of thermoset prepreg composites using out-of-autoclave technology	[14]
	A review on the out-of-autoclave process for composite manufacturing	[24]
Prepreg layup and defects	A review on the manufacturing defects of complex-shaped laminate in aircraft composite structures	[25]
	Automated material handling in composite manufacturing using pick-and-place systems—a review	[26]
	Prospects and challenges of nanomaterial engineered prepregs for improving interlaminar properties of laminated composites––a review	[27]
	A mini review on manufacturing defects and performance assessments of complex shape prepreg-based composites	[28]
Prepreg tack	Prepreg tack: A review of mechanisms, measurement, and manufacturing implication	[29]
Prepreg testing	Quality analysis and control strategies for epoxy resin and prepreg	[30]

**Table 3:** Recent studies published with information on different B-stage control strategies.

Study	B-Staging Conditions	References
Development of in-house unidirectional carbon/epoxy prepregs and its characterization for aerospace applications	9 h at room temperature	[37]
Development of a new structural prepreg: characterization of handling, drape, and tack properties	Varying the exposure time at room temperature	[65]
A novel custom-tailored epoxy prepreg formulation based on epoxy–amine dual-curable systems	Varying the amount of low-temperature curing agent	[66]
Influence of cure agent, treatment, and fiber content on the thermal behavior of a curaua/epoxy prepreg	38 hr at room temperature	[67]
Poly(amidoamine) functionalized graphene oxide incorporated carbon/epoxy prepreg composites for enhanced electrical and thermal Properties	30 min at 80 °C	[4]
Processing, thermal, and mechanical properties of composite laminates with natural fibers prepregs	24 hr at room temperature	[68]
Tack of epoxy resin films for aerospace-grade prepregs: Influence of resin formulation, B-staging, and toughening	15 min at 80 °C	[69]

**Table 4:** The chemical structure and physical properties of the resin, curing agents, and accelerator. Reproduced with permission [66]. Copyright © 2020 Elsevier.

Name	Chemical Structure	Physical Properties
DGEBA	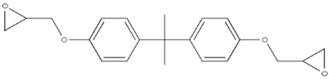	* EEW = 183–188 g/mol Density = 1.17 g/cm^3^
DETA	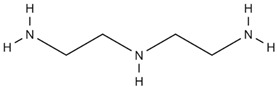	* HEW = 20.06 g/mol Molar mass = 103.17 g/mol
DICY	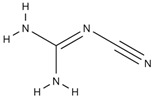	* HEW = 12.05 g/mol Melting point = 208–211 °C
DIURON	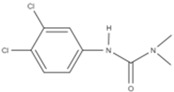	Melting point = 158 °C Molar mass = 233.09 g/mol

* EEW and HEW stand for Epoxy Equivalent Weight and Hydrogen Equivalent Weight.

**Table 5:** Intrinsic parameters that affect prepreg tack.

Factor	Description
Resin viscosity	Epoxy resin flowability
Prepreg architecture	Impregnation level, tack enhancing resin layers, resin types, resin volume fraction, etc.
Fiber volume fraction	Fiber/resin ratio
Degree of cure	B-stage cure of resin
